# Effects of age-related hearing loss and background noise on neuromagnetic activity from auditory cortex

**DOI:** 10.3389/fnsys.2014.00008

**Published:** 2014-01-31

**Authors:** Claude Alain, Anja Roye, Claire Salloum

**Affiliations:** ^1^Rotman Research Institute, Baycrest Centre for Geriatric CareToronto, ON, Canada; ^2^Department of Psychology, University of TorontoToronto, ON, Canada; ^3^Institute of Medical Sciences, University of TorontoToronto, ON, Canada

**Keywords:** aging, MEG, hearing loss, auditory cortex, inhibition (psychology)

## Abstract

Aging is often accompanied by hearing loss, which impacts how sounds are processed and represented along the ascending auditory pathways and within the auditory cortices. Here, we assess the impact of mild binaural hearing loss on the older adults’ ability to both process complex sounds embedded in noise and to segregate a mistuned harmonic in an otherwise periodic stimulus. We measured auditory evoked fields (AEFs) using magnetoencephalography while participants were presented with complex tones that had either all harmonics in tune or had the third harmonic mistuned by 4 or 16% of its original value. The tones (75 dB sound pressure level, SPL) were presented without, with low (45 dBA SPL), or with moderate (65 dBA SPL) Gaussian noise. For each participant, we modeled the AEFs with a pair of dipoles in the superior temporal plane. We then examined the effects of hearing loss and noise on the amplitude and latency of the resulting source waveforms. In the present study, results revealed that similar noise-induced increases in N1m were present in older adults with and without hearing loss. Our results also showed that the P1m amplitude was larger in the hearing impaired than in the normal-hearing adults. In addition, the object-related negativity (ORN) elicited by the mistuned harmonic was larger in hearing impaired listeners. The enhanced P1m and ORN amplitude in the hearing impaired older adults suggests that hearing loss increased neural excitability in auditory cortices, which could be related to deficits in inhibitory control.

## Introduction

Hearing abilities diminish with age largely due to changes that take place in the cochlea. However, there is increasing evidence suggesting that changes in the peripheral system alone cannot adequately account for all hearing problems encountered by older adults. Rather deficits in central auditory processing are also likely playing an important role (Martin and Jerger, 2005; Humes et al., 2012). Scalp recordings of auditory evoked potentials (AEPs) and auditory evoked fields (AEFs, the magnetic counterpart of AEPs) may be useful for differentiating an “aging” from a “hearing loss” basis for the auditory deficits observed in older adults. Furthermore, it may also help identify brain areas that are more susceptible to hearing loss and/or the aging process.

In healthy normal hearing adults, AEPs are usually composed of a positive, a negative, and then a positive wave that peak at about 50 (P1), 100 (N1), and 180 ms (P2) after sound onset, respectively. Converging evidence from lesion studies in humans (e.g., Woods et al., 1987; Alain et al., 1998), magnetoencephaplography (MEG) (e.g., Hari et al., 1980; Hari, 1991; Reite et al., 1994), and brain source modeling (e.g., Scherg and Von Cramon, 1986; Picton et al., 1999) are consistent with generators located in or near Heschl’s gyrus. The P1, N1, and P2 waves are mainly stimulus-driven (i.e., exogenous) responses thought to index signal detection (Hillyard et al., 1971). The amplitude and latency of these responses are influenced by the signal-to-noise ratio (Martin et al., 1997, 1999; Whiting et al., 1998; Martin and Stapells, 2005). Martin and colleagues, for example, examined the effects of competing signals by presenting speech signals embedded in noise (Martin et al., 1997; Martin and Stapells, 2005). They found that speech identification abilities decreased when exposed to poorer signal-to-noise ratios with the performance decrement paralleled by both increased N1 latencies and decreased N1 amplitudes. More importantly, the latency and amplitude of the N1 significantly correlated with behavioral assessments of signal detectability (Martin et al., 1997) with electrophysiological thresholds closely approximating behavioral thresholds (Lightfoot and Kennedy, 2006). These findings suggest that AEPs provide a sensitive measure of signal audibility, which may prove useful at evaluating the effects of age-related hearing loss on central auditory processing.

The effects of normal aging on AEPs and AEFs have been examined in numerous reports using a variety of paradigms and stimuli. In many studies, the P1 wave has been found to be larger in older adults than in younger adults. (e.g., Smith et al., 1980; Pekkonen et al., 1995; Bertoli et al., 2005; Kovacevic et al., 2005; Fabiani et al., 2006; Alain and Snyder, 2008; Ross and Tremblay, 2009; Soros et al., 2009; Ross et al., 2010; Lister et al., 2011; Alain et al., 2012) Similar age-related increases in the N1 amplitude have been reported (e.g., Anderer et al., 1996; Chao and Knight, 1997; Alain and Woods, 1999; Amenedo and Diaz, 1999; Harkrider et al., 2006; Ross and Tremblay, 2009; Soros et al., 2009), albeit with less consistency (for a failure to find age difference see, Pfefferbaum et al., 1980; Smith et al., 1980; Picton et al., 1984; Barrett et al., 1987; Iragui et al., 1993; Bertoli et al., 2002; Tremblay et al., 2004; Kovacevic et al., 2005; Lister et al., 2011). The effect of age on the P2 amplitude is more equivocal with some studies reporting no age difference (Ford et al., 1979; Picton et al., 1984; Barrett et al., 1987; Iragui et al., 1993; Tremblay et al., 2004) while others observing smaller (Goodin et al., 1978; Smith et al., 1980; Ross and Tremblay, 2009) or larger (Pfefferbaum et al., 1980; Ford and Pfefferbaum, 1991; Fabiani et al., 2006; Alain and Snyder, 2008) amplitudes in older adults. However, the effects of age on the P2 latency are more consistent, with most studies reporting an age-related increase in P2 latency (e.g., Goodin et al., 1978; Iragui et al., 1993; Tremblay et al., 2004; Alain and McDonald, 2007). Together these findings are often taken as evidence for an age-related change in central auditory processing. The implicit assumption is that the changes are specific to age rather than to other factors such as hearing loss. However, in most studies, the young and older adults do not only differ in terms of age, they often also differ in hearing thresholds. This potential confound is acknowledged in many studies and there have been some attempts to control for it, for example, by adjusting sound intensity using the mean audiometric thresholds (e.g., Ross et al., 2007) or by using hearing thresholds as a covariate (e.g., Alain and McDonald, 2007).

Another approach to separate the contribution of age and hearing loss on central auditory processing consists of comparing older normal-hearing adults with those who have mild or severe hearing loss. The few studies published so far using this approach have yielded unexpected and surprising results. For instance, Harkrider et al. (2006) compared young adults, older normal-hearing adults, and older adults with mild to moderate hearing loss. They found that the speech-evoked N1 wave was larger in hearing impaired than in normal-hearing older adults. Tremblay et al. (2003) also report a larger N1 in older adults with hearing impairment but only for a subset of speech sounds with voice onset time greater than 40 ms. Conversely, Bertoli et al. (2005) tested young adults, normal-hearing older adults, and hearing-impaired older adults. They found reduced N1 amplitudes to pure tone stimuli in hearing-impaired older adults compared to older normal hearing adults. Hence, it remains unclear whether hearing loss contributes to the age-related difference in N1 amplitude. The results for the P2 are slightly more consistent, with studies reporting no difference in P2 amplitude between normal-hearing and hearing-impaired older adults (Bertoli et al., 2005; Harkrider et al., 2006). In older adults, there is little evidence that hearing loss per se affects the P1, N1, or P2 latency (Tremblay et al., 2003; Bertoli et al., 2005; Harkrider et al., 2006).

The present study aims to further investigate the effect of hearing loss on central auditory processing using MEG. This study is an extension of an earlier study that examined the effects of age and background noise on listeners’ ability to process a mistuned harmonic in an otherwise harmonic complex tone (Alain et al., 2012). The use of harmonic complex sounds with and without a mistuned harmonic provide the means to assess age-related differences related to processing sound onset as well as neural activity associated with encoding frequency periodicity. The study from Alain et al. (2012) revealed an age-related increase in P1m (magnetic counterpart of the P1 wave from EEG) as well as a delayed P2m compared to young adults. The mistuned harmonic generated an object-related negativity (ORN), which was comparable in amplitude between young and older adults. The ORN is a relatively new event-related potential (ERP) component that has been associated with the perception of concurrent sound objects induced by the mistuning of a low tonal element of a harmonic complex tone (e.g., Alain et al., 2001, Alain et al., 2002; Hautus and Johnson, 2005). It is usually illustrated by subtracting the ERPs elicited by tuned from those elicited by mistuned stimuli. The ORN provides a metric to assess whether age and/or hearing loss impaired listeners’ ability to segregate concurrent sounds based on periodicity cues, which is important for understanding speech in noisy situations. If hearing loss plays an important role in the age-related difference in central auditory processing, as previously reported, then we should observe a group difference in neuromagnetic brain activity elicited by sound onset and mistuning between older adults with normal-hearing and those who are hearing-impaired.

## Materials and Methods

### Participants

A total of 36 older adults were recruited for the study. Based on their hearing status (pure tone thresholds, see below), they were categorized into one of two groups, normal hearing vs. mildly hearing impaired. All were screened to exclude health or mental problems and/or medications that might affect cognitive function or brain activity.

Participants were recruited from the local community and provided informed consent in accordance with the guidelines established by the University of Toronto and Baycrest Centre. Two participants were excluded due to excessive head motion during the MEG recording (one from each group). A final sample of 17 hearing impaired adults (range: 62–82; mean age = 70.6, standard deviation (s.d.) = 6.3; 8 men) were compared with a sample of 17 normal hearing adults (range: 63–76; mean age = 67.8, s.d. = 3.6; 9 men). The two groups did not differ in their mean age (*t*_(32)_ = 1.60; *P* = 0.12). All but one participant in each group were right handed.

### Hearing assessment

Our criteria for mild hearing loss were pure-tone thresholds greater than 25 decibel (dB) and hearing level (HL) for octave frequencies from 250 to 2000 Hz in both ears. Participants with pure-tone thresholds less than or equal to 25 dB hearing level were included in the normal hearing group. All participants completed a speech-in-noise (SIN) test. Four lists of six sentences were used from the Quick SIN (Etymotic Research, 2001; Killion et al., 2004) test. All sentences were spoken by a female in a background of four-talker “babble” at 70 dB sound pressure level (SPL). The babble in each list of sentences was increased in 5 dB steps in order to vary the signal-to-noise ratio (SNR) from 0 dB to +25 dB. Participants repeated back the target sentence. Each sentence included five “keywords”. A point was awarded for each key word of a possible total of five points per sentence. The SNR loss was determined by subtracting the total number of correct words from 25.5. This number represents the SNR required to correctly identify 50% of the sentences (Killion et al., 2004).

All hearing impaired participants filled in a brief hearing checklist (self-report questionnaire) that was based on the Revised American Academy of Otolaryngology-Head & Neck Surgery’s 5 min hearing test (Koike et al., 1994), to compare the amount of hearing loss with the hearing loss of other older adults.

### Stimuli and task

The stimuli used during the recording of neuromagnetic activity consisted of complex sounds created by combining ten pure tones of equal intensity (i.e., 200, 400, 600, 800, 1000, 1200, 1400, 1600, 1800, and 2000 Hz). The fundamental frequency ( *f*_0_) was 200 Hz and the third partial could either be tuned (i.e., 0% mistuning at 600 Hz) or mistuned upwards from its original value by 4% (to 624 Hz) or 16% (to 696 Hz). Stimuli were digitally generated at a sampling rate of 24,414 Hz using a System 3 Real-Time Processor (Tucker Davis Technologies, Alachua, FL) and presented binaurally via an OB 822 Clinical Audiometer using ER-3A transducers (Etymotic Research, Elk Grove, IL, USA) and reflectionless 2.5-m plastic tubes. All three stimulus types (0%, 4%, and 16% mistuning) had durations of 200 ms and rise/fall times of 5 ms. They were equiprobable and presented in random order with an inter-stimulus interval (ISI) that varied randomly between 800 and 1200 ms in 100 ms steps (rectangular distribution). All participants, regardless of their hearing status, were presented with stimuli at a fixed 75 dB SPL. There were three listening conditions corresponding to the background against which the stimuli were presented within a block of trials. That is, within the entire block of trials, the stimuli were either presented without background noise, or against a continuous low (45 dBA), or against a continuous moderate (65 dBA) level broadband Gaussian white noise. For each noise condition, participants were presented with a total of 300 trials (100 of each stimulus type: 0%, 4%, and 16% mistuning), for a grand total of 900 stimuli during the course of the experiment. The order of noise conditions was counterbalanced between participants. The intensity of the stimuli and the intensity of the noise were measured using a Larson-Davis SPL meter (Model 824, Provo, Utah). The plastic tubes from the ER-3A transducers were attached to a 2 cc coupler on an artificial ear (Model AEC100l) connected to the SPL meter. Separate measurements were taken for left and right ear channels.

All participants completed a behavioral task that was performed after the MEG recording in a double-walled sound attenuated chamber (IAC model 1204A, Electromedical Instruments, Mississauga, ON). The same stimuli were presented over Eartone ER-3A insert earphones using a System 3 Real Time Processor and a GSI 61 Clinical Audiometer. Following the presentation of each stimulus, participants were asked to indicate if they heard one sound (i.e., a buzz) or two sounds (i.e., a buzz plus another sound with a pure tone quality) by pressing two different buttons label as “1” or “2”, respectively. These responses (i.e., 1 or 2) reflected participants’ perceptions based on the mistuning manipulation. Responses were registered using a multi-button response box and the next stimulus was presented 1500 ms following the previous response. Participants did not receive feedback on their performance. Prior to the behavioral experiment, participants were presented with a sample of stimuli to familiarize themselves with the task and the response box. After this familiarization phase, participants completed six blocks of trials. For each noise condition, two blocks of 150 trials were presented for a total of 300 trials (100 of each stimulus type: 0%, 4%, and 16% mistuning). For each participant, we calculated the proportion of trials in which participants reported hearing two sounds as well as dprime (*d*′) and beta (β) values. For the calculation of *d*′ and β, trials whereby participants were presented with 0% mistuning and responded “2” were treated as “false alarms”, and trials whereby participants were presented with mistuned stimuli and responded “2” were treated as “hits” (Moore et al., 1986; Alain et al., 2012).

### Neuromagnetic recording and analysis

The MEG recording took place in a magnetically shielded room using a helmet shaped 151-channel whole cortex neuro-magnetometer (OMEGA, CTF Systems, VSM Medtech Inc., Vancouver, Canada). AEFs were recorded in a passive listening session as participants watched a muted, subtitled movie of their choice. This design allowed us to examine the impact of noise on cortical activity elicited by stimuli while minimizing the influence of top-down processes on AEF amplitude. The use of muted subtitled movies has been shown to effectively capture attention without interfering with auditory processing (Pettigrew et al., 2004). To minimize movement, participants laid down throughout the recording.

The neuromagnetic activity was recorded continuously with a sampling rate of 625 Hz and an on-line, low-pass filter with a cutoff frequency of 200 Hz. The analysis epoch included 200 ms of pre-stimulus activity and 600 ms of post-stimulus activity. The epochs were scanned for artifacts using Brain Electrical Source Analysis (BESA) software (version 5.2). To account for individual differences in the amplitude of neuromagnetic brain activity, the maximum intensity for accepting single epochs of the MEG signals was adjusted for each participant and ranged from 1515 to 6607 fT/cm. AEFs were averaged separately for each stimulus type and noise condition (i.e., block of trials). For normal hearing adults, the number of trials included in the single-subject grand average (i.e., all stimulus type and noise condition combined) ranged from 587 to 697 while for hearing impaired adults it ranged from 587 to 752. For each participant and for each noise condition, we computed the grand average of AEFs that comprised all stimulus types. This average was used to generate a dipole source model of the scalp recorded AEFs. The source waveforms for each experimental condition were computed from the resulting source model.

We used BESA software (version 5.2) for dipole source modeling. The analysis used the spherical head model of Sarvas (1987). Before dipole source modeling, the averaged data were low-pass filtered at 20 Hz (12 dB/octave; zero phase). First, we seeded a left and a right dipole in the temporal lobe near Heschl’s gyrus using a magnetic resonance imaging template from BESA. Then, we fitted the location and orientation of each dipole to account for a 40 ms interval centered on the peak of the N1m wave. We chose to model the N1m wave because it was the largest and most reliable deflection from the AEF elicited by the harmonic complex tones. The analysis was performed on the grand average across stimulus types to enhance signal-to-noise ratio and because the differences in source location between the N1m elicited by tuned and mistuned stimuli were expected to be small (Arnott et al., 2011). Peak amplitude and latency were determined as the largest positivity (P) or negativity (N) in the individual source waveforms during a specific interval. The measurement intervals were 30–90 ms (P1m), 70–160 ms (N1m), 150–260 ms (P2m), and 120–220 ms (ORN). AEF amplitude and latency were analyzed using a mixed model repeated measures ANOVA with hearing status (normal, impaired) as the between-groups factor and mistuning (0%, 4%, 16%), noise condition (no, low, moderate), and hemisphere (left, right) as the within-group factors. When appropriate, the degrees of freedom were adjusted with the Greenhouse-Geisser epsilon (ε) and all reported probability estimates are based on the reduced degrees of freedom, although the original degrees of freedom are reported. Bonferroni corrections were applied for all posthoc, pairwise comparisons. For the behavioral data, we performed mixed model repeated measures ANOVA with hearing status (normal, impaired) as the between-groups factor and mistuning (0%, 4%, 16%), and noise condition (no, low, moderate) as the within-group factors.

## Results

### Behavioral data

#### Pure tone thresholds

**Figure 1:**
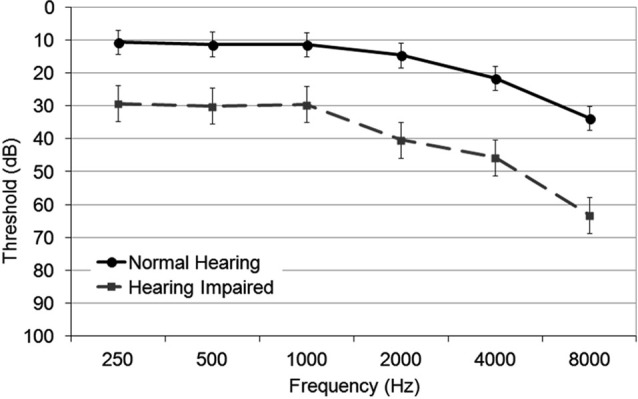
**Group mean audiometric thresholds in normal hearing and hearing impaired older adults for octave frequencies between 250 and 8000 Hz**. The error bars indicate standard error of the mean.

The two experimental groups significantly differed in pure tone thresholds (Figure 1) with normal hearing adults having lower average thresholds (*M* = 12.7 dB HL) than hearing impaired adults (*M* = 32.3 dB HL), *F*_(1,32)_ = 75.64, *P* < 0.001, *η*^2^ = 0.70) for 250–2000 Hz. Despite the difference, both normal hearing and hearing impaired groups showed typical age-related decline in pure tone thresholds, most prominent in the higher frequencies, *F*_(3,96)_ = 11.62, *P* < 0.001, *η*^2^ = 0.27. This decline was even more prominent in the hearing impaired group (interaction group × tone frequency, *F*_(3,96)_ = 3.62, *P* = 0.002, *η*^2^ = 0.01). There were no differences in hearing thresholds between the left and the right ear in either the normal hearing group or hearing impaired group (hemisphere *F* < 1; group × hemisphere *F* < 1; hemisphere × tone frequency, *F*_(3,96)_ = 1.48, *P* = 0.23).

#### Quick hearing check

According to the quick hearing check, all participants classified as hearing impaired reached scores that were within the lower 10 to 35% of other older adults classified as hearing impaired (Koike et al., 1994). Furthermore, the hearing impaired group performed worse in the Quick SIN test as revealed by a higher SNR loss (*M* = 4.21, *s.e.* = 0.72) compared to the normal hearing group (*M* = 1.90, *s.e.* = 0.64; *t*_(32)_ = −2.40, *P* = 0.02).

#### Behavioral task

**Figure 2:**
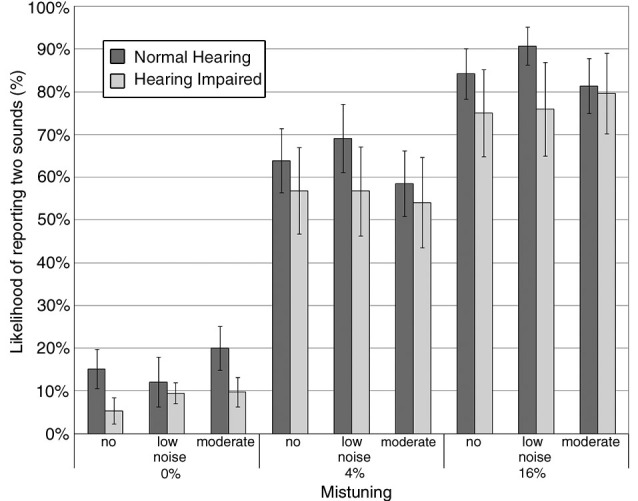
**Group mean likelyhood of reporting hearing two concurrent sounds as a function of mistuning and background noise**. The error bars indicate ±1 standard error of the mean. Normal hearing: darker gray bar; Hearing impaired: lighter gray bar.

Within the hearing impaired group, 5 of 17 participants did not finish the behavioral task and hence only 12 hearing impaired participants were included in the final analysis of the behavioral data. Overall, results showed that the proportion whereby participants indicated hearing two concurrent sounds increased with mistuning, *F*_(2,56)_ = 64.33, *P* < 0.001, *η*^2^ = 0.70, with the steepest slope for the low noise condition (interaction mistuning × noise, *F*_(4,112)_ = 2.23, *P* = 0.07, *η*^2^ = 0.07; Figure 2). The main effect of hearing status and noise was not significant, nor was the interaction between hearing status and noise. The three-way interaction between hearing status, noise and mistuning was not significant.

**Figure 3:**
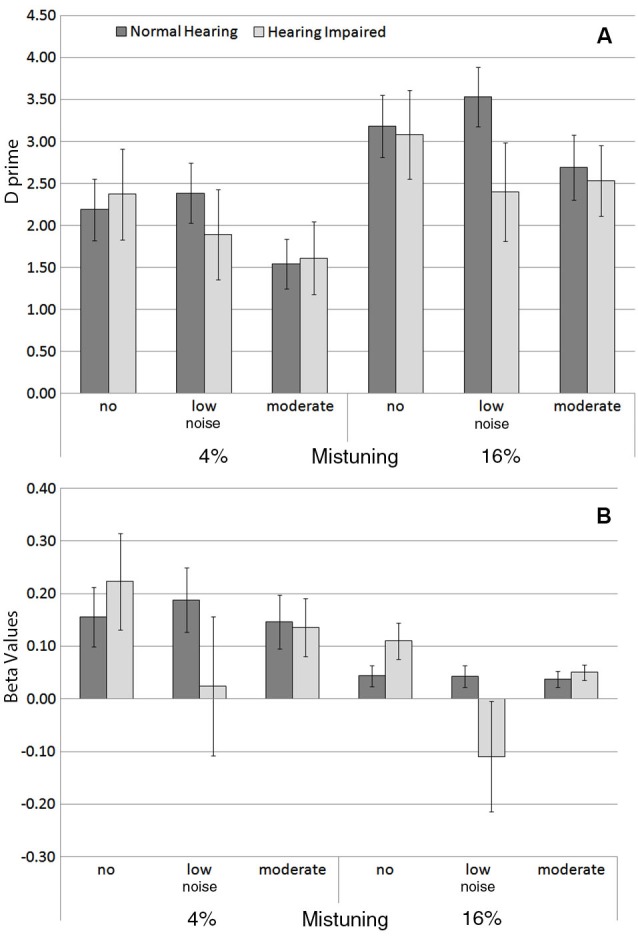
**(A)** Group mean sensitivity (*d′*) and **(B)** response bias (β) for each background noise condition for the 4% and for the 16% mistuned harmonics in normal hearing and in hearing impaired older adults. The error bars indicate ±1 standard error of the mean.

Figure 3 shows the group mean sensitivity (i.e., *d*′) and response bias (i.e., β) in hearing impaired older adults compared to normal hearing older adults in the three noise conditions. There was an increase in *d*′ with mistuning (Figure 3A; *F*_(1,27)_ = 14.23, *P* = 0.001, *η*^2^ = 0.34), but a decrease with increasing noise (*F*_(2,54)_ = 5.27, *P =* 0.008, *η*^2^ = 0.16). The main effect of hearing status was not significant, nor was the interaction between hearing status and mistuning or noise.

Overall, the response bias varied as a function of mistuning (Figure 3B), *F*_(1,27)_ = 5.03, *P* = 0.03, *η*^2^ = 0.16, with smaller β values for the 16% mistuning condition. However, there was no significant difference between normal hearing and hearing impaired older adults in β, nor was the interaction between hearing status and mistuning or hearing status and noise significant.

### Auditory evoked fields and dipole source location

**Figure 4:**
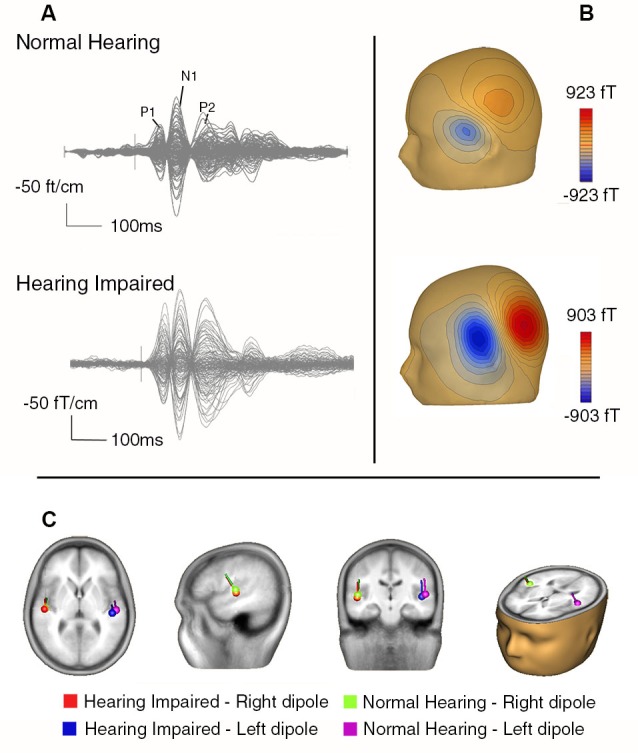
**(A)** Butterfly plot showing AEFs from a representative normal hearing older adult (top) and a hearing impaired older adult (bottom) averaged over all stimulus types from all sensors. **(B)** The contour maps for the N1m for these two participants indicate the direction of the magnetic flux (i.e., blue = negative, red = positive). **(C)** The group mean dipole location for the N1m from the normal and hearing impaired older adults overlaid on a magnetic resonance imaging template from BESA (5.3).

Figure 4 overlays the time course of the AEFs recorded with all MEG sensors averaged over the three noise conditions. The AEFs comprise an initial P1m peak at 68 ms followed by the larger N1m and P2m responses at 125 and 220 ms, respectively. The magnetic field topography at the latency of N1m for two representative participants is consistent with bilateral sources in the auditory cortices along the superior temporal gyrus.

The group mean locations for the N1m are also shown in Figure 4. The effects of hearing status and noise on N1m source locations were examined by comparing source coordinates (x, y, and z coordinates) separately. For the lateral-medial axis, the main effects of hearing status, noise, and hemisphere were not significant nor was the interaction between any of the factors. For the anterior-posterior axis, there was the typical main effect of hemisphere, *F*_(1,32)_ = 18.25, *P* < 0.001, *η*^2^ = 0.36), indicating that the source in the right hemisphere was more anterior than the one in the left hemisphere. The main effect of hearing status was also significant (*F*_(1,32)_ = 4.80, *P* = 0.04, *η*^2^ = 0.13), with more posterior N1 source locations for the hearing impaired group. There was also a main effect of noise (*F*_(2,64)_ = 6.84, *P* = 0.002, *η*^2^ = 0.18). Pairwise comparisons revealed that the N1m source location was more anterior under moderate noise conditions compared to no and low noise conditions (*P* < 0.05 in both cases). There was no difference in source location between the no and low noise conditions (*P* = 0.50).

For the inferior-superior axis, the hearing impaired group showed more inferior N1m source locations than the normal hearing group (*F*_(1,32)_ = 4.89, *P* = 0.03, *η*^2^ = 0.13). Furthermore, the N1m source was located more superior in the left hemisphere compared to the right hemisphere (*F*_(1,32)_ = 4.20, *P* = 0.049, *η*^2^ = 0.11). No other effect reached statistical significance.

### Source waveforms

**Figure 5:**
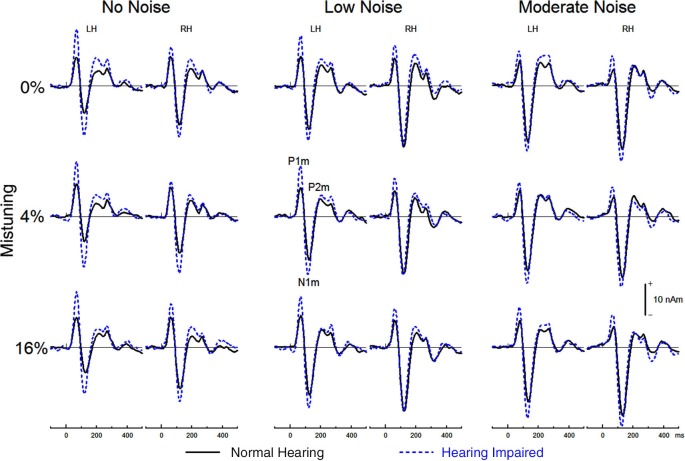
**Group mean source waveform from the left hemisphere (LH) and the right hemisphere (RH) for tuned (top) and mistuned stimuli**.

Figure 5 shows the group mean source waveforms in each hemisphere for hearing impaired and normal hearing older adults as well as for each stimulus type as a function of background noise. In both groups, the source waveforms comprised a P1m, N1m, and P2m peaking at about 68 ms, 125 ms, and 220 ms after sound onset.

#### Effects of hearing status, mistuning, and noise on auditory evoked field (AEF) latency

Analysis revealed no main effect of hearing status on P1m latency (*F* < 1). For both groups the P1m peaked earlier in the right (*M* = 66.00, *s.e.* = 1.47 ms) than in the left (*M* = 71.45, *s.e.* = 1.47 ms) hemisphere, *F*_(1,32)_ = 43.05, *P* < 0.001, *η*^2^ = 0.57. There was a main effect of noise such that stimuli embedded in moderate noise levels generated longer P1m latency compared to lower or no noise conditions, *F*_(2,64)_ = 8.75, *P* < 0.005, *η*^2^ = 0.21 (Table 1). The interaction between hearing status and noise conditions was significant (*F*_(2,64)_ = 4.36, *P* = 0.03, *η*^2^ = 0.15). P1m latency increased with noise only in normal hearing older adults (*F*_(2,32)_ = 13.03, *P* < 0.001). In hearing impaired older adults, the main effect of noise on P1m latency was not significant (*F* < 1). The main effect of mistuning was significant, *F*_(2,64)_ = 5.50, *P* = 0.007, *η*^2^ = 0.15. The 16% mistuned stimuli generated a longer P1m latency than the tuned or 4% mistuned stimuli (*P* < 0.05 in both cases). There was no difference in latency between the tuned and 4% mistuned stimuli nor did mistuning interact with any other factors.

**Table 1:** **Group mean P1m latency and amplitude**.

Group	Noise	Tuning	Latency (ms)	Std. Error	Amplitude (nAm)	Std. Error
**Normal Hearing**	No	0%	68.141	2.35	11.048	1.837
		4%	67.906	2.158	8.05	1.058
		16%	68.188	2.323	11.536	1.93
	Low	0%	65.976	2.097	10.405	1.621
		4%	66.165	2.256	10.095	1.51
		16%	66.824	2.361	10.882	1.624
	Moderate	0%	73.741	2.699	8.051	1.358
		4%	75.153	2.498	8.448	1.342
		16%	76.376	2.429	8.544	1.388
**Hearing Impaired**	No	0%	66.871	2.35	17.094	1.837
		4%	66.024	2.158	9.926	1.058
		16%	69.412	2.323	17.71	1.93
	Low	0%	66.447	2.097	15.972	1.621
		4%	66.400	2.256	16.136	1.51
		16%	67.906	2.361	16.199	1.624
	Moderate	0%	67.812	2.699	12.203	1.358
		4%	68.471	2.498	11.714	1.342
		16%	69.224	2.429	13.09	1.388

As with P1m, there was no main effect of hearing status on N1m peak latency, *F*_(1,32)_ = 1.78, *P* = 0.19, (Table 2) and the N1m peaked earlier in the right (*M* = 122.32, *s.e.* = 1.92 ms) than in the left (*M* = 124.96, *s.e.* = 1.84 ms) hemisphere, *F*_(1,32)_ = 6.13, *P* = 0.02, *η*^2^ = 0.16. N1m latency increased with increasing background noise levels, *F*_(2,64)_ = 28.62, *P* < 0.001, *η*^2^ = 0.47, and this effect of noise was more pronounced for normal hearing adults (noise × group interaction, *F*_(2,64)_ = 3.55, *P* = 0.04, *η*^2^ = 0.10). Furthermore, the effect of noise on the N1m latency was more pronounced in the left than in the right hemisphere (noise × hemisphere interaction, *F*_(2,64)_ = 3.59, *P* = 0.04, *η*^2^ = 0.10). The main effect of mistuning was significant, *F*_(2,64)_ = 2.36, *P* < 0.001, *η*^2^ = 0.13 such that the 16% mistuned stimuli generated a longer latency than the tuned or 4% mistuned stimuli (*P* < 0.001, in both cases). There was no difference between the tuned and 4% mistuned stimuli (*P* = 0.63).

**Table 2:** **Group mean N1m latency and amplitude**.

Group	Noise	Tuning	Latency (ms)	Std. Error	Amplitude (nAm)	Std. Error
**Normal Hearing**	No	0%	119.012	3.231	-12.564	2.776
		4%	118.494	3.157	-11.901	2.759
		16%	126.871	3.627	-12.786	2.759
	Low	0%	124.376	2.77	-18.623	3.423
		4%	124.329	3.008	-18.144	3.543
		16%	125.412	2.878	-19.56	3.564
	Moderate	0%	130.494	2.311	-20.521	2.821
		4%	131.859	2.226	-19.569	2.732
		16%	133.506	2.246	-21.065	2.791
**Hearing Impaired**	No	0%	117.647	3.231	-18.658	2.776
		4%	118.306	3.157	-19.197	2.759
		16%	123.718	3.627	-18.992	2.759
	Low	0%	117.459	2.77	-20.929	3.423
		4%	117.459	3.008	-21.279	3.543
		16%	122.306	2.878	-23.043	3.564
	Moderate	0%	123.153	2.311	-24.8	2.821
		4%	124.518	2.226	-24.351	2.732
		16%	126.588	2.246	-26.587	2.791

The effects of hearing status and experimental condition on the P2m latency are summarized in Table 3. As with the P1m and N1m, the P2m peak latency was unaffected by hearing status (*F* < 1). The P2m peaked earlier in the right (*M* = 210, *s.e.* = 4.3 ms) than in the left (*M* = 221, *s.e.* = 4.0 ms) hemisphere, *F*_(1,32)_ = 9.47, *P* = 0.004, *η*^2^ = 0.23. The main effect of noise level was significant, *F*_(2,64)_ = 11.86, *P* < 0.001, *η*^2^ = 0.27. Stimuli embedded in a moderate level of noise generated a longer P2m latency than those in the no noise or low noise condition (*P* < 0.001, in both cases). There was no difference in P2m latency between the no noise and the low noise conditions (*P* = 0.99). The main effect of mistuning was significant, *F*_(2,64)_ = 3.82, *P* < 0.03, *η*^2^ = 0.11. The 16% mistuned stimuli generated a longer latency than the tuned stimuli (*P* = 0.03). There was no significant difference between the 16% and the 4% mistuned stimuli (*P* = 0.11), nor was the difference between tuned and 4% mistuned stimuli significant (*P* = 0.99).

**Table 3:** **Group mean P2m latency and amplitude**.

Group	Noise	Tuning	Latency (ms)	Std. Error	Amplitude (nAm)	Std. Error
**Normal Hearing**	No	0%	213.176	7.239	7.335	1.294
		4%	203.247	6.706	6.914	1.292
		16%	216.424	6.755	6.121	1.411
	Low	0%	208.047	6.213	8.665	1.42
		4%	209.129	7.005	7.895	1.474
		16%	214.918	6.459	7.006	1.36
	Moderate	0%	220.329	5.321	8.317	1.363
		4%	219.859	5.402	8.151	1.337
		16%	229.412	5.612	6.642	1.263
**Hearing Impaired**	No	0%	209.176	7.239	11.469	1.294
		4%	215.388	6.706	10.014	1.292
		16%	214.306	6.755	9.659	1.411
	Low	0%	207.812	6.213	11.59	1.42
		4%	218.588	7.005	10.123	1.474
		16%	217.976	6.459	9.955	1.36
	Moderate	0%	224.141	5.321	11.116	1.363
		4%	218.588	5.402	8.793	1.337
		16%	224.518	5.612	8.987	1.263

To sum it up, the latency of the P1m, N1m, and P2m was little affected by hearing loss. The source waveforms elicited by harmonic complex tones peaked earlier in the right than in the left hemisphere. Background noise and mistuning increased the latency of source activity from the auditory cortex. This effect of background noise on the latency of source waveforms was greater in normal hearing older adults than hearing impaired older adults. The effect of mistuning on the latency of source waveforms was comparable in both groups.

#### Effects of hearing impairment and noise on auditory evoked field (AEF) amplitude

The main effect of hearing status on P1m amplitude was significant (*F*_(1,32)_ = 5.76, *P* = 0.02, *η*^2^ = 0.15), with hearing impaired older adults generating a larger P1m response (*M* = 14.45, *s.e.* = 1.41) than normal hearing older adults (*M* = 9.67, *s.e.* = 1.41). The main effect of noise was also significant, *F*_(2, 64)_ = 15.55, *P* < 0.001, *η*^2^ = 0.33). The P1m amplitude was smaller in the moderate noise condition than in the no noise or low noise conditions (*P* < 0.005, in both cases). There was no difference in P1m amplitude between the no noise and the low noise condition (*P* = 0.63). The interaction between hearing status and noise condition was not significant (*F*_(2,64)_ = 1.14, *P* = 0.32). There was a main effect of mistuning, *F*_(2,64)_ = 25.23, *P* < 0.001, *η*^2^ = 0.44 as well as a significant interaction between hearing status and mistuning (*F*_(2,64)_ = 3.71, *P* = 0.04, *η*^2^ = 0.10). Overall, the P1m was smaller for the 4% mistuned stimuli than for the tuned or the 16% mistuned stimuli (*P* < 0.001 in both cases) whereas the P1m generated by the 16% mistuned harmonic was larger than the one elicited by the tuned stimuli (*P* = 0.024). The interaction between hearing status and mistuning was due to greater changes in P1m as a function of harmonicity in older adults with mild hearing loss than older adults with normal hearing.

The ANOVA on the P1m amplitude also revealed a significant interaction between mistuning and noise (*F*_(4,128)_ = 22.09, *P* < 0.001, *η*^2^ = 0.41) and between hearing status, mistuning and noise (*F*_(4,128)_ = 3.96, *P* = 0.005, *η*^2^ = 0.11). Post hoc testing revealed that the effect of mistuning on P1m amplitude was actually present only in the no noise condition and that this effect was bigger for the hearing impaired group (no noise, main effect mistuning *F*_(2,64)_ = 34.83, *P* < 0.001; no noise, hearing status × mistuning *F*_(2,64)_ = 5.39, *P* = 0.007; no noise, effect of mistuning for hearing impaired *F*_(2,32)_ = 24.25, *P* < 0.001; no noise, effect of mistuning for normal hearing *F*_(2,32)_ = 10.61, *P* = 0.002; no significant effect of mistuning for low noise and moderate noise). Further, the interactions of noise by hemisphere (*F*_(2,62)_ = 7.59, *P* =0.002, *η*^2^ = 0.19), mistuning by hemisphere (*F*_(2,64)_ = 18.51, *P* < 0.001, *η*^2^ = 0.37), and mistuning by noise by hemisphere (*F*_(4,128)_ = 12.42, *P* < 0.001, *η*^2^ = 0.28) were significant.

The N1m amplitude was not significantly affected by hearing status (*F*_(1,32)_ = 1.46, *P* = 0.24). However, there was a main effect of noise (*F*_(2,64)_ = 14.84, *P* < 0.001, *η*^2^ = 0.32). The N1m was larger when stimuli were embedded in low or moderate background noise than when there was no background noise (*P* = 0.01 in both cases). There was no difference between the low and moderate noise condition (*P* = 0.27). The main effect of mistuning was also significant, (*F*_(2,64)_ = 6.74, *P* = 0.005, *η*^2^ = 0.17), and this effect is likely due to the ORN superimposed on the N1m thereby increasing its amplitude (see below). The interaction between hearing status and noise was not significant (*F* < 1) nor was the interaction between hearing status and mistuning (*F* < 1). The three-way interaction between hearing status, noise, and mistuning was not significant (*F* < 1). Lastly, the N1m amplitude was larger in the right than in the left hemisphere, *F*_(1,32)_ = 5.06, *P* = 0.03, *η*^2^ = 0.14.

For the P2m peak amplitude, the main effect of hearing loss was not significant (*F*_(1,32)_ = 2.61, *P* = 0.12) nor was the main effect of noise (*F* < 1), or the interaction between hearing status and noise (*F* < 1). The main effect of mistuning was significant (*F*_(2,64)_ = 17.29, *P* < 0.001, *η*^2^ = 0.35), with the P2m amplitude decreasing with the presence of mistuning. Pairwise comparison revealed smaller P2m for the 4% and 16% mistuned stimuli relative to tuned stimuli (*P* < 0.001, in both cases). The difference between 4% and 16% was not significant (*P* = 0.28). Further, the interaction between noise and hemisphere was significant (*F*_(2,64)_ = 6.15, *P* = 0.004, *η*^2^ = 0.16). However, none of the pairwise comparisons reached statistical significance. No other main effects or interactions reached significance.

#### Object-related negativity (ORN)

**Figure 6:**
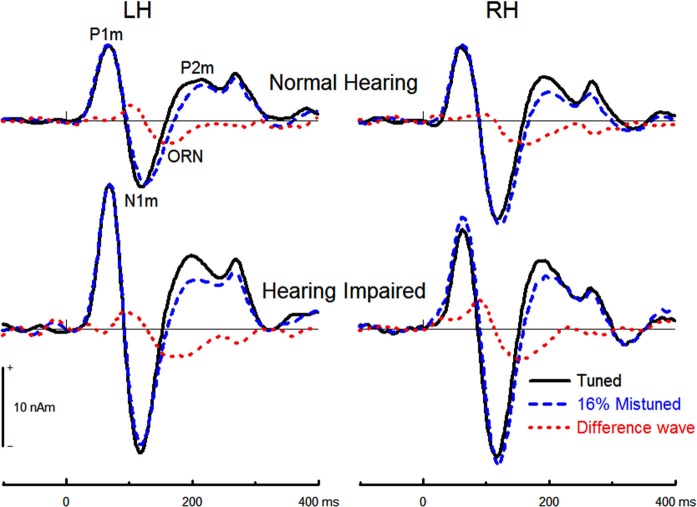
**Group mean source waveform for the tuned and the 16% mistuned stimuli and the corresponding difference in source waveforms**. The ORN peaked earlier when the harmonic was mistuned by 16% (*M* = 165, *s.e.* = 1.7 ms) than when it was mistuned by 4% (*M* = 175, *s.e.* = 1.7 ms), *F*_(1,32)_ = 18.71, *P* < 0.001, *η*^2^ = 0.37. The effect of hearing status was not significant (*F*_(1,32)_= 2.10, *P* = 0.16) nor was the effect of noise (*F* < 1) or hemisphere (*F*_(1,32)_ = 2.70, *P* = 0.11). The interaction between hearing status and mistuning was not significant (*F*_(1,32)_ = 2.01, *P* = 0.17) nor was the interaction between hearing status and noise (*F* < 1).

The effects of hearing status and noise on concurrent sound segregation are best illustrated in the difference waves between source waveforms for the tuned stimuli and for the 4% and the 16% mistuned stimuli (cf. Alain et al., 2001). For both groups, this difference wave showed an ORN that peaked at about 170 ms after sound onset (Figure 6).

The 16% mistuned harmonic stimuli generated a larger ORN amplitude than the 4% mistuned stimuli, *F*_(1,32)_ = 65.66, *P* < 0.001, *η*^2^ = 0.67. The effect of hearing status on the ORN peak amplitude was significant (*F*_(1,32)_ = 9.70, *P* = 0.004, *η*^2^ = 0.23), with hearing impaired older adults generating larger ORN than normal hearing older adults. The interaction between hearing status and mistuning was not significant (*F* < 1), nor was the three way interaction between group, mistuning and hemisphere (*F* < 1). No further main effects or interactions reached statistical significance.

## Discussion

The primary aim of this study was to assess the impact of mild hearing loss on cortical evoked responses in an effort to clarify age-related changes in auditory evoked responses. Specifically, this study examines whether these previously reported changes in the literature reflect age per se or whether they are partly due to age-related hearing loss. In the present study, there was a difference in pure tone thresholds of about 20 dB HL between older adults with normal hearing and those included in the mild hearing loss group. Moreover, older adults with mild hearing loss, as defined by pure tone thresholds, showed elevated thresholds for understanding speech in noise. Surprisingly, there was no significant difference between the two groups in the proportion of trials for which participants indicated hearing two concurrent sounds as a function of mistuning. That is, the likelihood of hearing the mistuned harmonic as a separate sound was little affected by mild hearing loss. Although this may appear surprising, the lack of differences between groups might be due to the drop out of bad performers during the behavioral part of the experiment in the hearing impaired group only. The lack of group difference could also be related to the forced choice procedure, which involved a subjective component and did not capture participants’ thresholds in detecting mistuning or parsing the mistuned harmonic as a separate tone.

### Effect of mild hearing loss on auditory evoked fields (AEFs)

In both groups, the source waveforms derived from modeling the N1m wave with bilateral dipoles in or near Heschl’s gyrus comprised a P1m, N1m, and P2m deflection. The N1m source location was more anterior and more inferior in the right than in the left hemisphere, and this is consistent with prior MEG studies (e.g., Pantev et al., 1998; Ross et al., 2010). These differences between left and right N1m sources map onto the anatomical asymmetry that showed a more anterior auditory cortex in the right than in the left hemisphere (Penhune et al., 1996; Leonard et al., 1998; Rademacher et al., 2001). The N1m source location was more posterior and more inferior in older adults with hearing impairment than without. This group difference was unexpected and may reflect neuroplastic changes in auditory cortices associated with peripheral hearing loss. For example, the changes in N1m source location could reflect the recruitment of cortical neurons that have lost their afferent input due to peripheral hearing loss (Dietrich et al., 2001). The recruitment of de-afferented neurons could also contribute to the increased amplitude of neuromagnetic responses.

The P1m amplitude was larger in older adults with mild hearing loss than in older adults with normal hearing, which suggests that aging is not the sole contributor to increased sensory evoked response amplitude. Hearing loss, therefore, plays an important role in modulating cortical evoked response amplitude. Furthermore, since stimuli and noise SPL levels were identical for all participants, sound intensity cannot account for increased P1m amplitude between the two groups. However, identical sound intensity may not have yielded the same loudness and it is possible that older adults with hearing impairment experienced greater loudness recruitment than older adults with normal hearing. Loudness recruitment refers to the perceptual phenomenon of sounds becoming rapidly louder with increasing sound levels and has been proposed to contribute to increased N1m amplitude in patients with hearing impairment (Morita et al., 2003).

Prior research examining the effect of hearing impairment on auditory evoked responses have observed larger N1 amplitudes in hearing impaired than in normal hearing adults (Morita et al., 2003; Tremblay et al., 2003; Harkrider et al., 2006). In the present study, the main effect of hearing status on N1m amplitude was not significant, despite the N1m source waveforms appearing slightly larger in the hearing impaired. The difference between the present study and prior work could be related to the material used. Prior research used speech sounds (Tremblay et al., 2003; Harkrider et al., 2006) or pure tones (Morita et al., 2003) with relatively long rise/fall times whereas we used complex sounds that had a well-defined and abrupt rise time. Consequently, our stimuli were more optimal to generate P1m and N1m responses whereas the material used in prior studies only generated a clear N1 response. Thus, our finding coupled with those of prior research provides converging evidence for increased neural excitability in primary and associative auditory cortices following sensory-neural hearing loss. Future experiments that manipulate the stimulus envelope is, however, needed to better understand the origin of these differences in the latency of the effect of hearing loss on cortical evoked responses.

In aging research, the enhanced amplitude of sensory evoked responses (i.e., P1 and N1 deflections) is often thought to index listeners’ difficulty in filtering out task-irrelevant information (Chao and Knight, 1997; Alain and Woods, 1999; West and Alain, 2000; Gazzaley et al., 2005), which has been related to a decline in prefrontal function (West, 1996; Chao and Knight, 1997; Alain and Woods, 1999). This account emphasizes the role of top-down control processes mediated by higher brain functions and assumes deficits in descending auditory pathways. Empirical support for this proposal includes studies in humans that showed increased amplitudes of middle latency auditory evoked responses following lesions to the dorsolateral prefrontal cortex (Knight et al., 1989; Alho et al., 1994). An alternative of this top-down account focuses on impaired inhibitory functions along the ascending auditory pathways as well as a loss of frequency selectivity. Evidence from animal studies has revealed a relationship between sensory-neural hearing loss and increased neural excitability within the ascending auditory pathway (Willott and Lu, 1982) and primary auditory cortex (Kotak et al., 2005), which likely reflects deficits in inhibition (Willott and Lu, 1982; Caspary et al., 1995, 2005; Kotak et al., 2005). It is worth noting that we observed effects of mild hearing loss primarily on early exogenous evoked responses. This suggests that sensory-neural hearing loss impacts early cortical registration of acoustic information and further research is needed to determine whether even earlier stages of cortical processing would be impacted by hearing loss and how this relates to auditory perception and attention.

As in our previous aging study, we also found that the ORN amplitude increased with increasing mistuning. Interestingly, the effect of mistuning on perception was comparable in older adults with or without mild hearing loss. However, the ORN elicited by the mistuned harmonic was enhanced in older adults with mild hearing loss compared to normal hearing older adults. The enhanced ORN amplitude in mildly hearing impaired older adults was unexpected and could be due to loudness recruitment and/or increased cortical reactivity. The effect of mild hearing loss on both transient onset responses and ORN provides converging evidence for a general decline in inhibitory processes along the ascending auditory pathway, which likely contributes to increased time-locked cortical activity.

### Effects of hearing loss and noise on the speed of auditory processing

The peak latency of AEFs indicates the amount of time taken to generate the neuromagnetic response after sound onset and provides a means for assessing speed of auditory processing. In the present study, the P1m latency was comparable for the two groups. This suggests that conduction time in the ascending auditory pathways may be little affected by mild hearing loss. The N1m and P2m latencies were also comparable in older adults with and without mild hearing loss. Our findings are consistent with those of earlier EEG studies using speech sounds (Tremblay et al., 2003; Harkrider et al., 2006), which also found that mild hearing loss had little impact on the latencies of the N1 and P2 waves. This is important as it suggests that mild hearing loss does not impact the speed of processing. In a prior study using the same stimuli and listening conditions (Alain et al., 2012), we found age-related increases in N1m and P2m latencies, which was consistent with earlier research (e.g.,Tremblay et al., 2002; Matilainen et al., 2010). The age-related increase in latency of auditory evoked responses may reflect a slowing in auditory processing and/or a broadening of the temporal integration window (Emmer et al., 2006; Gleich et al., 2007; Huang et al., 2009) with older adults reaching a saturation in the auditory evoked responses at a longer latency than young adults. The fact that the ORN latency was comparable between older adults with and without hearing loss suggests that mild hearing loss does not significantly delay the early computation needed to segregate the mistuned harmonic as a separate sound object.

Presenting auditory stimuli against moderate background noise increased latencies of P1m, N1m, and P2m deflections. The noise-related increase in latencies of exogenous components is consistent with prior studies using speech sounds (Whiting et al., 1998; Parbery-Clark et al., 2011). In the present study, the noise-related increase in P1m and N1m latencies was only present for the normal hearing group, suggesting that mild hearing loss and background noise do not independently affect central auditory processing. Further research is needed to characterize in more detail the impact of hearing loss on speed of processing and how it interacts with age.

### Noise-induced increase in N1m amplitude

Another goal of the present study was to assess whether the noise-induced increase in N1m amplitude previously reported for young adults would also be observed in older adults with mild hearing loss. For both groups, the N1m amplitude was larger when stimuli were embedded in low and moderate background noise than when there was no background noise. As such, this finding replicates and extends those of earlier studies (Alain et al., 2009, Alain et al., 2012) to older adults with mild hearing loss. This noise-induced increase in sensory evoked responses is not limited to harmonic complexes, but has also been observed for speech sounds in young adults (Shtyrov et al., 1999; Parbery-Clark et al., 2011) and children (Anderson et al., 2010). The increased N1 amplitude may be related to efferent feedback connections between the auditory cortex and the thalamus, inferior colliculus, and/or auditory brainstem nuclei, whose role would be to enhance the SNR in adverse listening situations (Alain et al., 2009). Such a proposal is supported by a positive correlation between the N1 amplitude for stimuli embedded in noise and the performance in a speech-in-noise task (Parbery-Clark et al., 2011). Another possibility is that low and moderate levels of background noise enhance states of arousal, which then similarly enhance the amplitude of sensory evoked responses to improve performance in working memory tasks (Han et al., 2013).

### Concluding remarks

The present study aimed to clarify the role of age-related hearing loss in the enhancement of cortical evoked responses previously reported in the literature. We found increased cortical evoked responses in older adults with mild hearing loss compared to age-matched controls that have clinically normal hearing. Our findings suggest that age-related decline in hearing sensitivity plays an important role in modulating the amplitude of auditory evoked responses. More importantly, our findings highlight the importance to control for age-related difference in hearing sensitivity while investigating the impact of aging on central auditory processing.

## Conflict of interest statement

The authors declare that the research was conducted in the absence of any commercial or financial relationships that could be construed as a potential conflict of interest.
